# Effect of vegan toothpastes on surface characteristics of human enamel after erosion-abrasion

**DOI:** 10.1590/0103-644020256086

**Published:** 2025-10-24

**Authors:** Maria Sylvia de Almeida Pessa, Raissa Manoel Garcia, Roberta Tarkany Basting, Fabiana Mantovani Gomes França, Taís Scaramucci, Cecilia Pedroso Turssi, Waldemir Francisco Vieira-Junior

**Affiliations:** 1 Departamento de Odontologia Restauradora, Faculdade São Leopoldo Mandic, Instituto de Pesquisas São Leopoldo Mandic, Campinas, SP, Brazil; 2 Departamento de Dentística, Faculdade de Odontologia, Universidade de São Paulo, São Paulo, SP, Brazil; 3 Departamento de Odontologia Restauradora, Faculdade de Odontologia de Piracicaba, Universidade de Campinas, Piracicaba, SP, Brazil

**Keywords:** Dental enamel, diet, vegan, feeding behavior, tooth erosion

## Abstract

The study evaluated the effect of vegan toothpaste on the surface and elementary constitution of enamel submitted to erosion-abrasion. Human enamel blocks (3x3mm) were submitted to *in vitro* erosion-abrasion cycling (1% citric acid, 4x/day). After the first and last erosive challenge, the blocks were brushed with the toothpaste: conventional fluoride-free (Bitufo), containing SnF_2_/SnCl_2_ (Oral-B Gingiva Detox, 1100 ppm F^-^), vegan fluoride-free (Boni Natural), or vegan with NaF (Colgate Zero, 1100 ppm F^-^). The control area was covered with tape, and the test area was left exposed. The tape was removed after cycling, and the blocks were evaluated for surface roughness (Ra, n=10), scanning electron microscopy (SEM, n=5), and energy dispersive spectroscopy (EDS, at%, n=5). Data analysis used split ANOVA, Kruskal-Wallis, and Dunn tests (α=0.05). Ra values in the test area were higher (*p*=0.01) for fluoride-free (0.389±0.09) and vegan fluoride-free (0.383±0.08) toothpastes compared to SnF_2_/SnCl_2_ (0.297±0.05) and fluoridated vegan (0.303±0.06) toothpastes. EDS analysis showed higher Na% (*p*=0.02) in enamel exposed to SnF_2_ and SnCl_2_ toothpaste (=0.7%) than vegan fluoride-free toothpaste (=0.5%). SEM images indicated less surface alteration in enamel exposed to SnF_2_/SnCl_2_ toothpaste. Vegan toothpaste with fluoride showed better performance in reducing the effects of surface alteration than vegan fluoride-free toothpaste; however, toothpaste containing SnF_2_/SnCl_2_ promoted the enamel surface to be less altered.

## Resumo

O estudo avaliou o efeito de dentifrícios veganos na constituição elementar e superfície do esmalte submetido à erosão-abrasão. Blocos de esmalte humano (3x3mm) foram submetidos à ciclagem *in vitro* de abrasão e erosão (ácido cítrico 1%, 4x/dia). Após o primeiro e último desafio erosivo, os blocos foram escovados com os dentifrícios: convencional sem flúor (Bitufo), contendo SnF_2_/SnCl_2_ (Oral-B Gingiva Detox, 1100 ppm F^-^), vegano sem flúor (Boni Natural), ou vegano com NaF (Colgate Zero, 1100 ppm F^-^). A área controle foi coberta com fita adesiva e a área teste foi deixada exposta. A fita foi removida após a ciclagem e os blocos foram avaliados quanto à rugosidade superficial (Ra, n=10), microscopia eletrônica de varredura (MEV, n=5) e espectroscopia de energia dispersiva (EDS, em %, n=5). A análise dos dados utilizou ANOVA, testes de Kruskal-Wallis e Dunn (α=0,05). Os valores de Ra na área teste foram maiores (*p*=0.01) para os dentifrícios sem flúor (0.389±0.09) e vegano sem flúor (0.383±0.08) comparado aos dentifrícios com SnF_2_/SnCl_2_ (0.297±0.05) e vegano com NaF (0.303±0.06). A análise de EDS mostrou maior % de Na (p=0,02) no esmalte exposto ao dentifrício contendo SnF_2_ e SnCl_2_ (=0,7%) do que no vegano sem flúor (=0,5%). Imagens de MEV indicaram menor alteração superficial no esmalte exposto ao dentifrício com SnF_2_/SnCl_2_. O dentifrício vegano com flúor apresentou maiores efeitos na redução da alteração de superfície do que o dentifrício vegano sem flúor; entretanto, o dentifrício contendo SnF_2_/SnCl_2_ possibilitou menores alterações de superfície.

**Figure:**
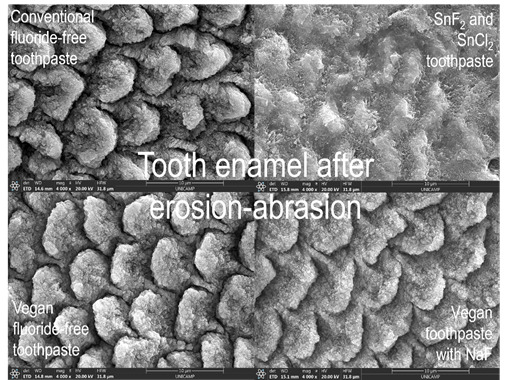

## Introduction

The search for more sustainable habits that minimize environmental damage has been increasing, and more people are concerned about the impact of their choices and habits worldwide. This has led to changes in the population’s diet and food intake, particularly in the growth of vegetarianism and veganism. It is estimated that the proportion of vegetarians/vegans worldwide ranges widely from 40% in India, the most representative country, to 1% in Portugal, 3.3% in the United States, and 14% in Brazil^1^. Overall, the main goal of this social framework is the consumption chain and not just that of limiting one’s food choices^2^.

The vegan movement is a philosophy and lifestyle that seeks to exclude, as much as practically possible, all forms of animal exploitation, whether for food, clothing, or any other purpose, and promote the development and use of animal-free alternatives ^2^. Products categorized as vegan are consumed not only by supporters of vegetarianism/veganism but also by a growing population keen on reducing environmental harm. To this end, studies are needed to characterize the effects of vegan or vegetarian oral hygiene products on dental tissues. 

Nutrition and diet are currently considered determinants of oral health ^3^, and there is an emergent awareness of the impact of food choice, nutrient intake, and patients' overall nutritional status on dental tissues ^3^. Eating habits can contribute to erosive tooth wear (ETW), and followers of a vegetarian diet may have twice as high risk for this condition as compared to non-followers of this type of diet ^4^. ETW is a non-carious loss of dental hard tissue due to acids; this wear is multifactorial and does not involve bacteria ^5^. It is mediated by biological factors, such as salivary calcium, phosphate and fluoride content, dental anatomy, buffering capacity, and salivary flow, and behavioral factors, such as lifestyle, eating habits, and oral hygiene ^6^.

However, sometimes, a patient may not notice the progression of this condition until it reaches advanced stages, at which point the tooth becomes so worn that it may result in unpleasant symptoms, such as dentin hypersensitivity and aged dentition ^6^. Consequently, dentists are encouraged to seek early signs of ETW in their patients to implement preventive measures to control the condition ^6^. Diet is the most widely studied etiological factor in ETW since excessive and frequent consumption of acidic drinks and foods has been described as a decisive contributing factor ^6^. Repeated contact with acidic substances associated with brushing could cause a topographical change in the tooth surface and lead to a structural loss ^7^.

A vegan lifestyle concerns ETW development since a vegan diet may consume more acidic foods, such as fruits, juices, marinades, and soluble vitamin C tablets ^4^. It should be pointed out that more acid-based vegan ingredients can replace animal-derived ingredients in different foods; therefore, individuals who pursue a vegan diet more frequently throughout the day may increase the number of acid challenges. Based on diet, adults with an omnivorous diet may be associated with a greater risk of periodontal problems and dental caries, while vegetarians/vegans may be associated with a greater risk of ETW ^3^. 

Veganism also affects the choices of oral hygiene products, which often do not contain any active principles like fluoride in their formulation. This calls for attention since toothpastes are the primary vehicle for fluoride delivery in most populations ^8^. Fluoride also plays an important role in reducing damage to enamel caused by erosion ^9^. Sodium fluoride (NaF) can promote the formation of a layer like CaF_2_ on tooth surfaces to provide first-line protection against acids, and the association between fluoride and other elements, such as ion stannous, can form acid-resistant precipitates on the tooth surface or can be incorporated into the surface to make it less soluble ^10^. In view of the absence of studies characterizing vegan oral care products and their impact on hard dental tissues, this study aimed to evaluate the effect of vegan toothpaste on the surface and the elementary constitution of human enamel subjected to an erosive-abrasive model. The null hypotheses tested where the tested toothpaste would not present any differences 1- in tooth enamel roughness or 2- in the elemental composition of the surface. 

## Material and methods

### Study Design

The present study was *in vitro* and presented blocks of human dental enamel as experimental units. The following factors were under study: I) toothpaste (4 levels): conventional without fluoride (Bitufo, Cotia, São Paulo, SP, Brazil), with stannous fluoride (Oral-B Gingiva Detox Deep Clean, Procter & Gamble, Louveira, SP, Brazil), vegan without fluoride (Mint and Turmeric, Boni Brazil, São Paulo, SP, Brazil), and vegan with sodium fluoride (Colgate Zero, Colgate-Palmolive, São Paulo, SP, Brazil); II) surface areas analyzed (2 levels): control (sound enamel) and test (exposed to erosive-abrasive cycling). The following variables were investigated: roughness (Ra, n = 10), energy dispersive spectroscopy (at%, n = 5), and scanning electron microscopy (qualitative, n = 5). 

### Group division

In this study, two conventional-type toothpastes were evaluated, one without fluoride and one with stannous fluoride and stannous chloride (SnF_2_ and SnCl_2_), and two vegan toothpastes, one without fluoride and the other with fluoride (NaF). The composition, active agents, vegan characterization, pH analysis (MPA 210, MS Tecnopon Instrumentação, Piracicaba, Brazil), and toothpaste batch are presented in Table 1. 

**Table 1:** Description of toothpastes

Toothpaste (Manufacturer)	Composition (pH)	Fluoride	Vegan^1^	Lot
Bitufo (Bitufo, Coty, São Paulo, SP, Brazil)	Hydrated silica, sorbitol, water, glycerin, xylitol, PEG-8, sodium lauryl sulfate, aroma, cellulose gum, xanthan gum, flower extract, calendula flower extract, chamomile flower extract, saccharin sodium, melissa flower extract, titanium dioxide - ^2^pH = 5.76	Without	No	L2060AS
Oral-B Gengiva Detox-Deep Clean (Procter & Gamble, Louveira, SP, Brazil)	Silica, water, sorbitol, silica, sodium lauryl sulfate, carrageenan, sodium gluconate, aroma, xanthan gum, zinc citrate, tin chloride, tin fluoride, sodium saccharin, sodium hydroxide, Cl 77891, limonene, sucralose - ^2^pH = 6.30	Association (1100 ppm): Tin fluoride and tin chloride	No	L1212GR
Boni Natural Mint and Turmeric (Boni Brasil, São Paulo, SP, Brazil)	Calcium carbonate, hydrated silica, water, glycerin, sodium lauryl sarcosine, xanthan gum, spearmint (mentha viridis) leaf oil, mint arvensis leaf oil, benzyl alcohol, marigold flower extract, peppermint, citrus, aurantifolia oil, sodium benzoate, melaleuca, citrus grandis peel oil, melaleuca alternifolia (tea tree), sucralose, turmeric, potassium sorbate - ^2^pH = 7.93	Without	Yes	L.184/21-1
Colgate Zero (Colgate-Palmolive, São Paulo, SP, Brazil)	Sorbitol, water, hydrated silica, xylitol, sodium lauryl sulfate, aroma, PEG-12, cellulose gum, benzyl alcohol, stevia, rebaudiana extract, rebaudioside, limonene - ^2^pH = 6.70	Sodium fluoride (1100 ppm)	Yes	NS0C03557-20C

*The compositions presented are those provided by the manufacturers.* ^*1*^ *The manufacturer may indicate through advertising that the product is vegan or present a vegan certificate seal indicating that the product does not contain ingredients of animal origin or use animals at any stage of its production or testing.* ^*2*^ *The pH of the solubilized toothpaste (1:3) was determined in triplicate using a pH meter.*

### Specimen preparation

Forty healthy human molars were used, without carious lesions, cracks, or restorations, and were collected after submission and ethical approval of the project by the local ethics committee (CAAE: 53875621.0.0000.5374). The teeth were stored in a refrigerated environment at 4°C until the beginning of the experimental procedures ^6^. Then, the teeth were cleaned with periodontal curettes (Duflex, SS White, Rio de Janeiro, RJ, Brazil) and pumice (SS White, Rio de Janeiro, RJ, Brazil) with water at low speed, followed by washing with distilled water. The specimens were obtained from the middle third of the buccal surface of the teeth. They are cut to the dimensions of 3 mm x 3 mm x 1.5 mm, then embedded in polyester resin using a silicone mold, and polished in a polishing machine (Buehler, Ecomet 250, Bluff, IL, USA) with 400-, 600- and 1200-grit paper, and polishing cloths with 0.3 µm diamond spray (AROTEC, Cotia, SP, Brazil).

The lateral surface of the specimens was covered with unplasticized polyvinyl chloride (UPVC) adhesive tape (Chartpak Graphic Art Tape-BG1252M, Chartpak, Leeds, MA, USA), used to separate the control from the test area ^11^ and to allow analysis of the enamel's surface structure after the erosive-abrasive cycling. After placing the tape, the specimens were randomized into study groups at n=10. 

### Erosion-abrasion cycling

The erosive challenge ^12^ ^,^ ^13^ was performed with 1% citric acid solution (pH = 2.6, natural pH) for 5 min with agitation at 100 rpm (SK 0330-Pro, Dragon Laboratory Instruments, Beijing, China) and room temperature, followed by immersion in artificial saliva (methyl-p-hydroxybenzoate, 2·00 g/L; sodium carboxymethyl cellulose, 10·0 g/L; KCl, 0·625 g/L; MgCl_2_·6H_2_O, 0·059 g/L; CaCl_2_·2H_2_O, 0·166 g/L; K_2_HPO_4_, 0·804 g/L; KH_2_PO_4_, 0·326 g/L), with pH adjusted to 6.75 using KOH ^14^, for 60 min. This procedure was repeated 4 times a day for 5 days. Toothbrushing was carried out twice a day with an electric toothbrush (Oral-b 35 PRO 2000, Schwalbach am Taunus, Germany - 15 s, 200 g of force corresponding to 1-3N; the pressure was controlled by the sensor contained on the toothbrush), in between the first and the last periods of exposure to artificial saliva. Toothpaste slurries were prepared before each abrasive challenge at 1:3 (w/w) dilution of the toothpaste tested (according to the experimental group), and the specimens were exposed to the slurries for 120 s. 

The same operator performed all the procedures throughout the experiment. After 24 hours of cycling, the specimens were kept refrigerated overnight and moistened with distilled water. After 5 days, the tape was removed, and roughness analysis, scanning electron microscopy, and energy dispersive spectroscopy were performed. 

### Surface roughness (Ra)

Ra analysis was performed using a surface profile measuring device (Surfitest SJ-210, Mitutoyo, Kanagawa, Japan). The readings were made at three different equidistant positions for each specimen in both the test and control areas, which were covered with tape. The average roughness (Ra, µm) of the specimen was obtained by averaging the readings. The equipment was configured using the following parameters: cut-off = 0.25, speed of 0.25 mm/s, and reading length x 3 (0.75 mm). 

### Energy-dispersive X-ray spectrometry (EDS) and Scanning electron microscopy (SEM)

After surface roughness analysis, half of the specimens were randomized for EDS (n=5) and SEM (n=5) analysis. After dissection for 48 h, an EDS device (Thermo Fisher Scientific UltraDry, ANAX-60P-B, Brno, Czech Republic) was used for qualitative and semiquantitative analysis of the chemical elements contained in the specimen ^15^. To this end, the specimens (n=5) were fixed in a specimen holder with carbon tape and aluminum tape for electrical contact and taken directly to the equipment for analysis under a current of 0.14 nA, spot size of 3, and 1000x magnification. The atomic percentage (at%) was obtained in the central region of the specimen (test area). The Ca/P ratio was also determined. 

SEM analysis (Thermo Fisher Scientific, Quattro S, Leo 440i, Cambridge, England) was used to observe the surface characteristics of the enamel in the central region of all the specimens. The specimens were immersed in increasing concentrations of ethanol, dissected for 48 h, and then (n=5) metalized with gold (Au layer thickness = 200 Aº, Sputter Coater EMITECH, K450, Kent, UK) to obtain images that would enable reading by electron beam scanning. The images were obtained using SEM, with a magnification of 1000x and 4000]x, under a voltage (kV) of 20, a current of 36 pA, and a spot size of 2. 

### Statistical Analysis

The results were subjected to exploratory analysis using the R program (R Core Team, R Foundation for Statistical Computing, Vienna, Austria). The Ra values were subjected to Analysis of Variance (ANOVA) in a split-plot scheme, considering the relationship between the test and control areas. The at% of the chemical elements (EDS) was analyzed using the Kruskal-Wallis and Dunn tests. All the tests were used, considering a significance level of 5%. 

For roughness, a sample size of 10 specimens per group (n=10), totaling 40 specimens, provided a test power of 80% (β=0.20) for minimum effect sizes of f=0.48 (large) for toothpaste, f=0.23 (medium) for cycling, and f=0.28 (medium) for the interaction between them, according to Cohen's criterion ^16^. For EDS (at%), a sample size of 5 specimens per group (n=5), totaling 20 specimens, provided a test power of 80% (β=0.20) for a minimum effect size of f=0.83 (large). Both analyses considered a significance level of 5% (α=0.05).

## Results

The Ra results are presented in Table 2. Regardless of the toothpaste, the Ra was significantly higher on the side exposed to the erosive-abrasive cycle than on the control side (p < 0.0001). In the test area, Ra was greater in the fluoride-free and vegan toothpaste without fluoride than in SnF_2_ and SnCl_2_ and vegan groups with fluoride (p = 0.0233). 

**Table 2:** Mean (Standard Deviation) of roughness values (Ra, µm) depending on the toothpaste and cycling.

	Erosive-abrasive cycling	
Toothpaste	Control area	Test area
Without fluoride	0.105 (0.030) Ba	0.389 (0.089) Aa
SnF_2_ and SnCl_2_	0.103 (0.025) Ba	0.297 (0.049) Ab
Vegan without fluoride	0.117 (0.045) Ba	0.383 (0.085) Aa
Vegan with fluoride (NaF)	0.110 (0.038) Ba	0.303 (0.058) Ab

*Different letters (uppercase letters horizontally and lowercase letters vertically) indicate statistically significant differences (p≤0.05). p(toothpaste)=0.0233; p(cycling)<0.0001; p(interaction)=0.0139.*

**Figure 1:**
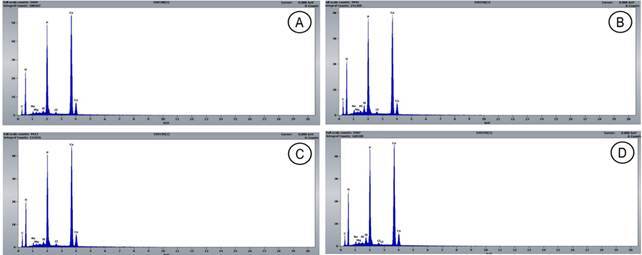
Graphs representing element quantification (EDS) of enamel according to the toothpaste. The toothpaste tested were: without fluoride (A), with SnF_2_ and SnCl_2_ (B), vegan without fluoride (C), and vegan with fluoride - NaF (D)

The results of the quantification of chemical elements are presented in Table 3. The following elements were found in the specimens: C, O, Na, Mg, Al, Si, Cl, Ca, and P. The toothpaste with SnF_2_ and SnCl_2_ presented a higher amount of Na than the fluoride-free vegan toothpaste (p =0.02). Based on the graphs (Figure 1), the presence of aluminum can be noticed only in the groups with SnF_2_ and SnCl_2_ and in the vegan toothpaste with fluoride (NaF). 

**Figure 2:**
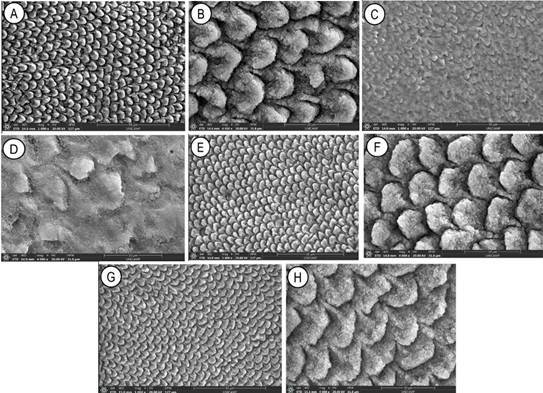
Images of toothpaste without fluoride (A) and (B), with SnF_2_ and SnCl_2_ (C) and (D), vegan without fluoride (E) and (F), and vegan with fluoride (G) and (H), using SEM; magnifications of 1000× (A, C, E, and G), and 4000× (B, D, F, and H).

The images obtained in SEM are shown in Figure 2. Fluoride-free (Figures 2A and 2B) and fluoride-free vegan (Figures 2E and 2F) toothpaste produced a similar surface pattern with generalized interprismatic dissolution, including porosities, changes in the enamel prisms, and depressions (Figure 2E). The vegan toothpaste with fluoride (NaF) (Figures 2G and 2H) also demonstrated a pattern of interprismatic loss, but with a less deep appearance and with maintenance of the hydroxyapatite prism. The toothpaste with SnF_2_ and SnCl_2_ (Figures 2C and 2D) presented an altered surface, but without evident interprismatic dissolution and with surface coverage by a compound with inorganic characteristics. 

**Table 3:** Median (minimum values; maximum values) of the % of the elements according to the toothpaste.

	Toothpaste				
Element	Without fluoride	SnF_2_ and SnCl_2_	Vegan without fluoride	Vegan with fluoride (NaF)	p-value
O	60.5 (59.2; 63.8) A	60.2 (58.7; 60.9) A	59.5 (53.8; 61.7) A	61.3 (59.6; 61.9) A	0.45
Ca	18.9 (15.9; 21.0) A	19.2 (15.3; 20.0) A	18.0 (16.5; 19.5) A	16.9 (15.2; 19.6) A	0.69
P	11.6 (11.0; 12.8) A	12.1 (10.4; 12.3) A	11.5 (10.0; 11.8) A	10.9 (10.2; 11.9) A	0.49
C	7.2 (6.1; 9.1) A	8.7 (7.1; 11.4) A	8.7 (5.9; 14.2) A	9.0 (6.1; 13.2) A	0.54
Na	0.6 (0.6; 0.7) AB	0.7 (0.6; 0.8) A	0.5 (0.4; 0.6) B	0.6 (0.4; 0.7) AB	0.02
Cl	0.2 (0.2; 0.3) A	0.2 (0.2; 0.3) A	0.3 (0.2; 0.3) A	0.2 (0.2; 0.2) A	0.31
Si	0.2 (0.0; 2.1) A	0.0 (0.0; 1.9) A	0.4 (0.1; 4.7) A	0.87 (0.1; 1.7) A	0.58
Mg	0.1 (0.0; 0.2) A	0.1 (0.1; 0.1) A	0.1 (0.0; 0.2) A	0.1 (0.0; 0.2) A	0.82
Al	0.0 (0.0; 0.0) A	0.0 (0.0; 0.1) A	0.0 (0.0; 0.0) A	0.0 (0.0; 0.01) A	0.90
Ca/P ratio	1.6 (1.4; 1.6) A	1.6 (1.5; 1.6) A	1.6 (1.5; 1.7) A	1.51 (1.5; 1.6) A	0.45

*Legend: Distinct horizontal letters indicate statistically significant differences (p≤0.05).*

## Discussion

Vegan and vegetarian diets are considered economically viable, sufficiently practicable, sustainable, and appropriate for protecting the environment, thus reducing pollution and minimizing global climate impacts ^2^. However, one nutrition impacts oral health, and an individual's health status affects nutrient acquisition and use ^3^ ^,^ ^4^. In this sense, a vegetarian diet may be associated with a greater risk of ETW ^4^. The absence of fluoride in the formulation of toothpaste also influences damage to enamel from ETW ^9^. Thus, the present study aimed to evaluate the effects of vegan toothpaste on the enamel surface. Based on the results, the null hypotheses were rejected. 

Regardless of their vegan characterization, the toothpaste that promoted the least change in Ra was the fluoridated ones. Fluoride protects the surface against acid attacks and favors the remineralization process ^7^. Conventional fluorides, such as the NaF found in Colgate Zero toothpaste (vegan-fluoride toothpaste), rely on the formation of calcium fluoride (CaF_2_) deposits ^17^. SnF_2_ (Oral-B Gengiva Detox-Deep Clean) is considered an agent with an anti-erosive potential ^7^ ^,^ ^18^ since it is not only deposited on the surface but also incorporated into the enamel and dentin ^17^. The present study's findings corroborate with this since this active ingredient caused a less altered enamel surface (Figure 2).

Although no Ra-related differences were found between SnF_2_/SnCl_2_ and NaF fluoridated toothpaste, in the present study, SEM images showed little or no mineral deposit barrier when the vegan toothpaste with NaF was evaluated. In this respect, a solution composed of distilled water and NaF promotes no precipitation on enamel after 2 min exposure ^19^. In contrast, the toothpaste with SnF_2_ and SnCl_2_ presented images suggestive of mineral deposition and a less altered surface layer, thus suggesting an acid-resistant layer from incorporating stannous in three distinct forms in the crystal, as previously reported ^20^. 

Furthermore, not only SnF_2_ but also SnCl_2_ is capable of depositing stable amounts of tin on enamel ^18^. However, stannous peaks were not found in the EDS analysis. These would be more significant in the presence of proteins such as albumin and mucin ^21^; however, in the present *in vitro* study, mucin- and albumin-free artificial saliva were used. Other methodologies commonly used to perform ETW analysis of dental substrates include optical profilometry, contact profilometry, and/or a rugosimeter ^8^ ^,^ ^22^. The comparison of roughness values between the control area and the test area may indicate a change in the topography of the enamel due to the dissolution and structural alteration of the hydroxyapatite crystals. In this respect, and as previously reported ^23^, the exposure to fluoridated agents resulted in lower roughness values for the eroded enamel, as found in the fluoridated toothpaste used in the present study. As observed in a previous study, the herbal extracts commonly contained in both vegan toothpastes used in the present study can increase roughness ^24^. This can be seen particularly in the fluoride-free vegan toothpaste, which has different plant-based active ingredients in its composition (Table 1).

*In vitro* and *in situ* erosion-abrasion models can provide effects of severe erosion or high-frequency brushing as a way of accelerating the process of enamel wear, which may often take years to occur in a clinical situation, mainly because the modifying biological factors cannot be simulated ^8^. Thus, artificial saliva was treated to simulate the oral environment and replicate some effects *in vivo*. The methyl p-hydroxybenzoate and sodium carboxymethyl cellulose increase the viscosity of saliva, simulating the mucin and protein content of natural saliva. At the same time, the other ingredients provide the inorganic components at levels comparable to those of natural saliva ^14^.

Considering the EDS results, the % of Ca and P and the Ca/P ratio of enamel surface were not different among the groups. This finding can be attributed to the pattern of dissolution and structural loss promoted by the erosive-abrasive challenge since total removal of the hydroxyapatite crystal could expose another crystal beneath it with a similar elemental composition. Other studies did not determine the differences in the elemental % of Ca and P when simulating erosion or abrasion since elemental percentages depended on the remineralizing agent used ^8^ ^,^ ^23^. The higher %Na and Na peaks found in the enamel exposed to the toothpaste with SnF_2_ and SnCl_2_, compared with the vegan toothpaste without fluoride, may have occurred due to the incorporation of Na into the surface precipitates. The Na may have been available in the toothpaste components of sulfate sodium, sodium gluconate, and sodium saccharin. 

In this respect, Al peaks were detected only in the fluoridated toothpastes. Al and F are elements with synergistic effects, such as reducing demineralization ^25^. Therefore, it is important to highlight that the study's limitations, inherent to in vitro investigations, include the lack of dentin evaluation and the absence of human saliva use. Thus, new studies have been developed using other wear analysis methodologies, such as dentin analysis, toothpaste abrasiveness, and RDA analysis, since patients who practice vegetarianism and veganism may have both enamel and dentin wear. 

Considering the prevalence of dental erosion and a previously suggested association with vegan and vegetarian diets ^4^, the clinical relevance of this study centers on the recommendation that toothpaste formulated for vegan patients should contain fluoride. Furthermore, based on the current study, which examines Brazilian vegan toothpaste, the SnF_2_/SnCl_2_ formulation does not bear a vegan certification. While these findings are important, they should be validated in situ and in vivo models to support clinical decision-making. Consequently, vegan toothpaste containing NaF was more effective in reducing enamel roughness than vegan toothpaste without fluoride. However, the toothpaste containing SnF_2_ and SnCl_2_ resulted in a less altered enamel surface.
